# A matter of timing

**DOI:** 10.7554/eLife.99181

**Published:** 2024-06-04

**Authors:** Zhiqi Tian, Jiajie Diao

**Affiliations:** 1 Department of Cancer Biology, University of Cincinnati College of Medicine Cincinnati United States

**Keywords:** SNARE, autophagy, autophagosome, phosphatidylinositol 4-phosphate, membrane charge, syntaxin 17, Human, Mouse

## Abstract

A change in the electric charge of autophagosome membranes controls the recruitment of SNARE proteins to ensure that membrane fusion occurs at the right time during autophagy.

**Related research article** Shinoda S, Sakai Y, Matsui T, Uematsu M, Koyama-Honda I, Sakamaki J, Yamamoto H, Mizushima N. 2024. Syntaxin 17 recruitment to mature autophagosomes is temporally regulated by PI4P accumulation. *eLife* **12**:RP92189. doi: 10.7554/eLife.92189.

Organelles perform a wide range of roles within cells, and many of these involve two organelles merging to form a single structure. Since most organelles are enclosed within a membrane, these mergers usually involve a complicated process called membrane fusion. A family of proteins known as SNARE proteins start the process of fusion by acting like a zip and pulling the two membranes together (Sutton et al., 1998).

Membrane fusion is important for a process called autophagy, which is used to recycle old, damaged or unneeded cellular components. Specifically, double membranes surround the material to be recycled, forming a closed ‘autophagosome’ that can fuse with an organelle called a lysosome. This allows digestive enzymes from the lysosome to enter the autophagosome and break down the contents, which can then be reused elsewhere in the cell.

Autophagy fusion is mediated by three members of the SNARE protein family; syntaxin17 and SNAP29 are initially found in the cytosol before being recruited to the membrane of the autophagosome to form a complex with VAMP7/8 on the lysosome (Itakura et al., 2012). The SNARE proteins then spontaneously zip together from their N-terminus (on the cytoplasm side of the membrane) to the C-terminus within the membrane. However, it is important that this process only occurs on mature, closed autophagosomes, and not on intermediate ‘open’ stages, otherwise the contents of the lysosome might leak into the cytosol. Accurately directing the different SNARE proteins to their target membranes at the right time is therefore crucial.

The leading candidates for directing syntaxin17 – the SNARE protein that is inserted into mature autophagosomes – are proteins known as RGM and LC3/GABARAP (Kumar et al., 2018). Additionally, a protein called filamin A has been shown to interact with and help recruit phosphorylated syntaxin17 to autophagosomes (Wang et al., 2023). However, these protein-protein interactions do not explain how syntaxin17 recruitment is precisely controlled in time. Now, in eLife, Noboru Mizushima (University of Tokyo) and colleagues – including Saori Shinoda (Tokyo and Kyoto Sangyo University) as first author – report that electric charges play a role in ensuring that syntaxin17 is recruited to autophagosomes at the right time (Figure 1; Shinoda et al., 2024).

**Figure 1.**
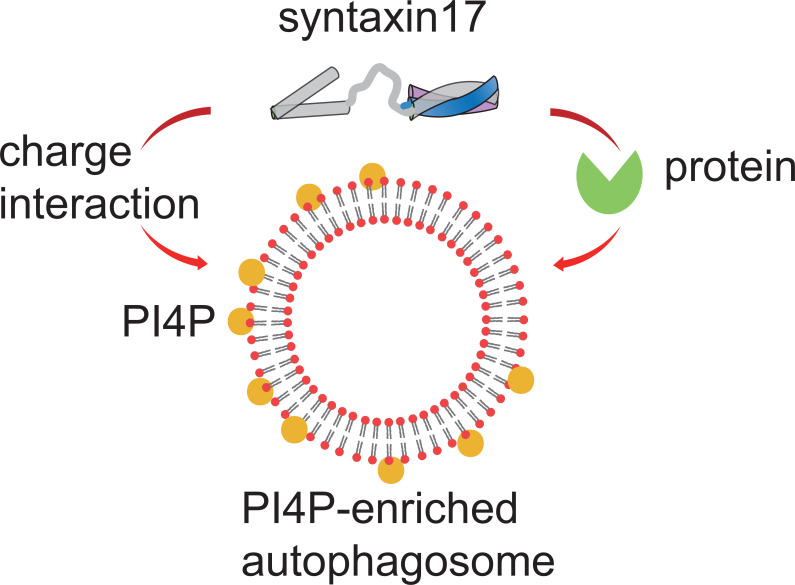
Regulating the recruitment of syntaxin17 to mature autophagosomes. When the membrane of an autophagosome (red circles with black lines) becomes enriched with PI4P (yellow circles), it becomes negatively charged. This negative charge recruits the positively charged C-terminus of a SNARE protein called syntaxin17 to the membrane. Syntaxin17 can also be recruited through interactions with chaperone proteins (green). PI4P: phosphatidylinositol 4-phosphate. SNARE: soluble N-ethylmaleimide-sensitive factor attachment protein receptors.

The team – who are also based at Tokyo, Kyoto University, Nippon Medical School and Cornell University – first showed that syntaxin17 recruitment to mature autophagosomes relies on its C-terminus being positively charged. Monitoring the membrane charges of autophagosomes at different stages of the formation process showed that, compared to an unclosed intermediate structure, mature autophagosomes are more negatively charged. This is mainly due to a phospholipid called PI4P accumulating in the membrane of the autophagosome as it matures. When PI4P was disrupted, syntaxin17 did not associate with the membrane. Moreover, computer simulations indicated that PI4P is also required for the insertion of syntaxin17 into the membrane. This suggests that the difference in the electric charge on a mature autophagosome and the charge on the C-terminal region of syntaxin17 drives the recruitment and insertion of syntaxin17 to the membrane. Collectively, these findings present a new model for how syntaxin17 is recruited to mature autophagosomes, which is also supported by a recent study from an independent group (Laczkó-Dobos et al., 2024).

Previous research has primarily focused on how membrane characteristics like charge, composition and curvature affect SNARE-mediated membrane fusion (Wickner, 2010). The pioneering findings of Shinoda et al. demonstrate the role of membrane charges in guiding the recruitment and insertion of SNARE proteins – critical steps that are initiated long before fusion occurs. Aside from protein chaperones, the findings of Shinoda et al. present a simple yet sophisticated model of SNARE recruitment based on membrane surface charges. Indeed, the observation that the surface charge of autophagosomal membranes changes during their formation represents a paradigm shift. Since PI4P is an important signaling molecule for cellular regulation (Tan and Brill, 2014), the accumulation of negatively charged PI4P on closed autophagosomes suggests a potential new regulatory mechanism for other autophagy proteins. This significant observation opens a new avenue for the future investigations on autophagy.

The work by Shinoda et al. underscores the multidisciplinary nature of contemporary cell biology research. As the factors influencing cellular processes now extend beyond traditional cell biology, studying them requires a broader array of technical tools. For instance, Shinoda et al. employed a computational technique called molecular dynamics simulation to illustrate the insertion of syntaxin17. And we have employed a combination of super-resolution imaging and biomechanical calculations in our own work on mitochondria (Chen et al., 2023). With contributions from experts in other fields such as physics, mechanics, and computer science, we can anticipate a continuing trend of significant and multidisciplinary discoveries in this area.
